# Serum Creatinine/Cystatin C-Based Muscle Indices for Sarcopenia and Graft Failure After Living Donor Liver Transplantation

**DOI:** 10.3390/jcm15176889

**Published:** 2026-09-05

**Authors:** Sa-Jin Kang, Yan Zhen Jin, Yu Sub Sung, Hyeun-Joon Bae, Kyoung-Sun Kim, Hye-Mee Kwon, In-Gu Jun, Jun-Gol Song, Gyu-Sam Hwang

**Affiliations:** 1 Department of Anesthesiology and Pain Medicine, Asan Medical Center, University of Ulsan College of Medicine, 88 Olympic-ro 43-gil, Songpa-gu, Seoul 05505, Republic of Korea; sajinkg12@gmail.com (S.-J.K.); yanzhen9051@163.com (Y.Z.J.); joshpheonix1@gmail.com (H.-J.B.); kyoungsun.kim@amc.seoul.kr (K.-S.K.); hyemee.kwon@amc.seoul.kr (H.-M.K.); igjun@amc.seoul.kr (I.-G.J.); kshwang@amc.seoul.kr (G.-S.H.); 2 Clinical Research Center, Asan Medical Center, University of Ulsan College of Medicine, 88 Olympic-ro 43-gil, Songpa-gu, Seoul 05505, Republic of Korea; asmilez.sung@gmail.com

**Keywords:** sarcopenia, skeletal muscle index, graft failure, living donor liver transplantation

## Abstract

**Background/Objectives**: Sarcopenia adversely affects outcomes after liver transplantation. The CT-based skeletal muscle index (SMI) is a validated prognostic measure but requires dedicated image analysis. We evaluated the diagnostic performance of creatinine/cystatin C-based serum muscle indices for identifying CT-defined sarcopenia and their prognostic value for graft failure in living donor liver transplantation (LDLT) recipients. **Methods**: We retrospectively analyzed 802 LDLT recipients (January 2023–April 2026). Using serum creatinine- and cystatin C-based equations, we calculated total body muscle mass (TBMM), appendicular skeletal muscle mass (ASM), and predicted SMI, and derived height-squared-normalized indices (TBMMI, ASMI); the creatinine-to-cystatin C ratio served as a non-equation-based comparator. CT-defined sarcopenia was classified using sex-specific reference cutoffs (males < 39.9, females < 28.9 cm^2^/m^2^). Diagnostic accuracy was assessed with sex-stratified ROC curves and DeLong test. The primary outcome was graft failure (recipient death or re-transplantation); its association with the best-performing index at 180 days, 1 year, and 3 years was evaluated using multivariable Cox models. **Results**: TBMMI showed the strongest correlation with SMI (*r* = 0.75). TBMMI discriminated CT-defined sarcopenia well (AUC 0.845 in males, 0.828 in females). After multivariable adjustment, each 1 kg/m^2^ decrease in TBMMI was independently associated with graft failure at 180 days (adjusted hazard ratio [HR] 1.42, 95% CI, 1.14–1.78), 1 year (HR 1.28, 1.05–1.56), and 3 years (HR 1.20, 1.02–1.40) (all *p* < 0.05). **Conclusions**: TBMMI is an accessible, non-imaging surrogate for SMI in LDLT recipients, and a lower TBMMI is independently associated with postoperative graft failure.

## 1. Introduction

Sarcopenia is defined as the age-related loss of skeletal muscle mass, accompanied by a decline in muscle strength and/or reduced physical performance [1]. Sarcopenia ultimately leads to severe adverse outcomes, including functional decline, increased morbidity and mortality, and substantial healthcare costs [2]. In chronic liver disease, muscle depletion occurs due to hyperammonemia, malnutrition, hormonal imbalances, endotoxemia, and decreased mobility [3]. Sarcopenia is highly prevalent in patients with cirrhosis, affecting approximately 40% to 48% of cases, and is independently associated with higher mortality [4,5,6,7]. Importantly, this prognostic impact is independent of the Model for End-Stage Liver Disease (MELD) score, suggesting that sarcopenia reflects aspects of physiological reserve and frailty not captured by the hepatic and renal parameters that constitute MELD [4,5,7,8]. Furthermore, integrating sarcopenia into the MELD score—resulting in the MELD–Sarcopenia score—has been shown to improve mortality prediction in cirrhotic patients [9], hepatocellular carcinoma patients awaiting liver transplantation (LT) [10], and cirrhotic patients following a transjugular intrahepatic portosystemic shunt placement [11], as well as predicting short-term readmission in patients with hepatic encephalopathy [12].

Identifying sarcopenia requires the direct quantification of muscle mass. Body composition can be assessed using dual-energy X-ray absorptiometry, bioelectrical impedance analysis (BIA), or by cross-sectional imaging, and current consensus definitions incorporate these measures of muscle quantity [1,2]. In LT, the accepted reference standard is the skeletal muscle index (SMI), which is calculated from the cross-sectional skeletal muscle area at the third lumbar vertebra (L3) on computed tomography (CT) and normalized to height squared [13,14]. Using L3 SMI, CT-defined sarcopenia has been independently associated with graft failure and mortality after living donor LT (LDLT) [15,16]. However, deriving SMI from CT requires specialized segmentation of the L3 section, posing a practical barrier to its routine use [17]. Therefore, non-imaging surrogates obtainable from routine laboratory testing have been explored [18].

Serum creatinine is primarily derived from skeletal muscle, whereas cystatin C is produced by all nucleated cells and is relatively independent of muscle mass [19]. Combining these two markers isolates the muscle-derived component of creatinine and can serve as a surrogate for muscle mass [20,21,22]. In the context of liver disease, the creatinine-to-cystatin C ratio has been studied as a simple surrogate marker for muscle mass; specifically, it is associated with sarcopenia and survival in patients with hepatitis C-related cirrhosis [23] and has been incorporated into waitlist mortality prediction scores for liver transplant candidates [17]. Recently, equation-based indices that convert serum creatinine and cystatin C into a direct estimate of muscle mass, rather than a relative ratio, have been evaluated as sarcopenia screening tools. In metabolic dysfunction-associated steatotic liver disease, a creatinine–cystatin C-based calculated body muscle mass discriminated low skeletal muscle mass defined by BIA better than the creatinine-to-cystatin C ratio alone [24]. However, whether a cystatin C-based muscle index, validated against SMI, is associated with post-LT outcomes in LT recipients has not yet been established.

Two knowledge gaps remain. First, it is unclear which creatinine/cystatin C-based index best reflects SMI, the reference standard for sarcopenia in LT. Second, previous studies have focused primarily on cross-sectional identification of low muscle mass without evaluating post-transplant outcomes. We therefore compared the diagnostic accuracy of these indices for CT-defined sarcopenia in adult LDLT recipients and examined whether the best-performing index was independently associated with graft failure and mortality.

## 2. Materials and Methods

### 2.1. Study Design and Population

**Figure 1:**
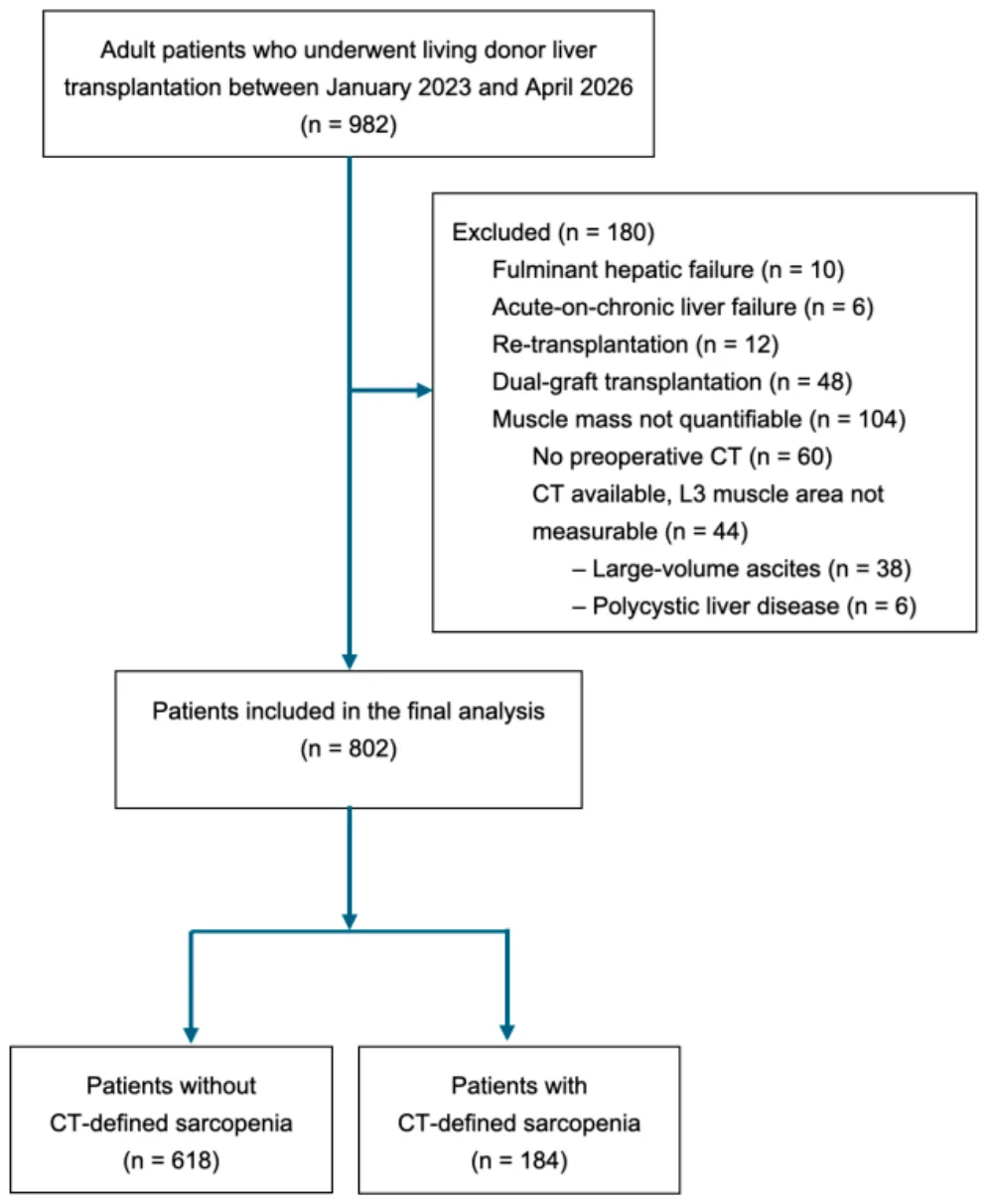
Flow diagram of patient selection. Abbreviations: CT, computed tomography.

The study was conducted in accordance with the Declaration of Helsinki and approved by the Institutional Review Board of Asan Medical Center. We retrospectively reviewed the medical records of adult patients (≥18 years) who underwent LDLT at Asan Medical Center between January 2023 and April 2026 (*n* = 982). Patients were excluded based on the following criteria: fulminant hepatic failure (*n* = 10), acute-on-chronic liver failure (*n* = 6), re-transplantation (*n* = 12), and dual-graft transplantation (*n* = 48). Recipients in whom muscle mass could not be quantified were additionally excluded (*n* = 104): no preoperative contrast-enhanced CT was available in 60, and in 44 a CT was available but the skeletal muscle area at the third lumbar vertebra could not be segmented, owing to large-volume ascites obscuring the muscle–fat boundary in 38 and polycystic liver disease in 6. After these exclusions, a total of 802 recipients were included in the final analysis (Figure 1). The requirement for informed consent was waived by the Institutional Review Board due to the retrospective nature of the study.

### 2.2. CT-Based Skeletal Muscle Assessment

Consistent with the North American Expert Opinion Statement on Sarcopenia in Liver Transplantation, we defined sarcopenia using the L3 SMI based on CT scans performed during the transplant evaluation work-up [13]. Preoperative abdominal CT scans were used to measure the cross-sectional skeletal muscle area at the level of the L3 inferior endplate. On the selected axial image, the skeletal muscle area—including the psoas, paraspinal, transversus abdominis, rectus abdominis, quadratus lumborum, and internal and external oblique muscles—was delineated using a predefined attenuation threshold of −29 to +150 Hounsfield units (HU) with semi-automated ImageJ-based segmentation software (AsanJ-Morphometry™, build 20250528; Asan Image Metrics, Seoul, Republic of Korea; https://aim-aicro.com/software/morphometry, accessed on 1 September 2025). Image segmentation was performed by Y.Z.J., with software operation supported by Y.S.S. The muscle area was normalized to height squared to derive the SMI (cm^2^/m^2^). Sarcopenia was defined a priori using sex-specific cutoffs corresponding to −2 standard deviations below the mean of a healthy young living-donor reference population (males < 39.9 cm^2^/m^2^, females < 28.9 cm^2^/m^2^), consistent with prior studies [2,15].

### 2.3. Serum Creatinine/Cystatin C-Based Muscle Indices

Using the preoperative serum creatinine (Cr), cystatin C (CysC), and hemoglobin (Hb) values measured closest to transplantation, along with patient age, height, and body weight (BW), we calculated three muscle mass estimates based on previously reported equations [25,26,27]. CysC is routinely measured at our center as part of the pre-induction laboratory assessment. Additionally, as a simple non-equation-based comparator, we used the creatinine-to-cystatin C ratio (CCR = Cr/CysC, reported as a unitless ratio).

Total body muscle mass (TBMM), equivalent to the previously described calculated body muscle mass [24], was estimated using the equation developed by Kim et al. [25]:

TBMM = (BW × Cr)/(k × BW × CysC + Cr),(1)

where k = 0.00675 for males and 0.01006 for females. Units: BW (kg), Cr (mg/dL), CysC (mg/L), TBMM (kg).

Appendicular skeletal muscle mass (ASM) was estimated using the equation developed by Shi et al. [26]:

ASM = 0.595 × 0.998^Age^ × 0.825^Female^ × 1.079^Black^ × 1.005^Height^ × BW^0.677^ × Cr^0.143^ × CysC^−0.104^, (2)

with the ethnicity term set to the non-Black reference group for this Korean cohort. “Female” is coded as 1 for females and 0 for males, and “Black” is coded as 1 for Black individuals and 0 for all other races. Units: Age (years), Height (cm), BW (kg), Cr (mg/dL), CysC (mg/L), ASM (kg).

Predicted SMI (pSMI) was estimated using the sex-specific equations described by Kusunoki et al. [27]:

pSMI (males) = 4.17 − 0.012 × Age + 1.24 × (Cr/CysC) − 0.0513 × Hb + 0.0598 × BW, (3)

pSMI (females) = 3.55 − 0.00765 × Age + 0.852 × (Cr/CysC) − 0.0627 × Hb + 0.0614 × BW. (4)

Units: Age (years), Cr (mg/dL), CysC (mg/L), Hb (g/dL), BW (kg), pSMI (kg/m^2^).

Because SMI is expressed per height squared, TBMM and ASM were correspondingly indexed to height squared to yield the TBMM index (TBMMI = TBMM/height^2^, kg/m^2^) and the ASM index (ASMI = ASM/height^2^, kg/m^2^), whereas pSMI is inherently expressed as an index. These four serum indices (TBMMI, ASMI, pSMI, and CCR) were subsequently evaluated as candidate surrogates for SMI.

### 2.4. Outcomes

The primary outcome was graft failure, defined as recipient death or re-transplantation from any cause. All-cause mortality was analyzed as a secondary outcome. The index date for all time-to-event analyses was the date of transplantation. For graft failure, recipients were followed until death or re-transplantation, whichever occurred first. For all-cause mortality, recipients were followed until death from any cause; re-transplantation was not considered a mortality event or a censoring event. All event-free recipients were administratively censored on 30 June 2026, the end date of the study period. Each outcome was analyzed with follow-up administratively truncated at 180 days, 1 year, and 3 years.

### 2.5. Statistical Analysis

For continuous variables, normality was assessed with the Shapiro–Wilk test; all continuous variables were non-normally distributed and are presented as median [interquartile range]. Categorical variables are presented as number (%). Baseline characteristics were compared between recipients with and without CT-defined sarcopenia using the Mann–Whitney U test for continuous variables, and the chi-square test or Fisher exact test for categorical variables. To assess selection bias, baseline characteristics and outcomes of the 802 analyzed recipients and the 104 excluded were additionally compared.

The association between each serum index and SMI was evaluated using Pearson correlation coefficients. Because both the estimating equations and the CT thresholds are sex-specific, correlations were also examined separately in men and women.

Diagnostic accuracy for identifying CT-defined sarcopenia was quantified using the area under the receiver operating characteristic curve (AUC) with DeLong 95% confidence intervals (CIs), calculated separately for males and females. AUCs were compared between indices using the DeLong test. Optimal cutoffs were determined by the Youden index. To assess the internal validity of the diagnostic performance of TBMMI, we performed bootstrap resampling with 1000 iterations in each sex, drawing samples of the same size as the original cohort with replacement. In each iteration, the AUC and the optimal cutoff maximizing the Youden index were re-estimated. Optimism was quantified as the mean difference between performance in the bootstrap sample and performance in the original cohort—for the Youden index, by applying the bootstrap-derived cutoff back to the original data—and was subtracted from the apparent estimate to yield the optimism-corrected AUC and Youden index.

To determine whether the serum muscle indices contributed information beyond simple anthropometry, we specified an anthropometric reference model containing age, sex, and body mass index (BMI). For SMI, we fitted linear regression models and compared the coefficient of determination (R^2^) of the reference model with that of the reference model extended by each index; the increment in R^2^ was tested with the incremental F test. For CT-defined sarcopenia, logistic regression models were fitted separately in men and women using age and BMI as the reference model, and discrimination was quantified as the AUC of the predicted probabilities. Increments in the AUC relative to the reference model were tested with the DeLong test for two correlated curves.

For the outcome analyses, follow-up duration was summarized using the reverse Kaplan–Meier method and is reported as the median with its 95% confidence interval. Graft-failure-free survival and patient survival were estimated by the Kaplan–Meier method, and the number of recipients at risk and the cumulative number of events were tabulated at 180 days, 1 year, and 3 years.

The best-performing index for identifying CT-defined sarcopenia—defined a priori as the index with the highest AUC—was primarily modeled as a continuous variable, with hazard ratios expressed per 1 kg/m^2^ decrease. Cox proportional hazards models were used to estimate the association between TBMMI and each outcome. The primary multivariable model used a prespecified covariate set comprising TBMMI, recipient age, recipient sex, the MELD-Na (Model for End-Stage Liver Disease–Sodium) score, the graft-to-recipient weight ratio, and intraoperative massive transfusion (≥10 units of red blood cells transfused during surgery).

Two sensitivity analyses were performed using alternative covariate specifications. First, an extended model additionally included hypertension, diabetes mellitus, donor age, and donor sex. Second, backward elimination was applied to the extended covariate set using the step function in R with the Akaike information criterion (AIC; k = 2); at each step the covariate whose removal produced the largest reduction in AIC was dropped, and elimination stopped when no further removal reduced AIC.

We also examined whether renal function or the severity of liver disease—both of which influence Cr and CysC—modified the association between TBMMI and graft failure in subgroup analyses. Subgroups were defined by preoperative renal function (estimated glomerular filtration rate [eGFR] < 60 vs. ≥60 mL/min/1.73 m^2^, calculated with the 2021 CKD-EPI creatinine equation) and by liver disease severity (MELD-Na < 15 vs. ≥15). Subgroup-specific hazard ratios were obtained from a single Cox model containing a multiplicative interaction term between TBMMI and the subgroup variable, with the prespecified covariates retained, and the interaction was tested by likelihood-ratio test.

Model assumptions were checked. The linearity of continuous covariates was evaluated by comparing each linear term with a natural cubic spline using a likelihood ratio test. The proportional hazards assumption was examined using scaled Schoenfeld residuals with a Kaplan–Meier time transform. Because the proportional hazards assumption was not satisfied for TBMMI, Cox models with follow-up truncated at 180 days, 1 year, and 3 years were used to summarize its association with each outcome over different follow-up windows. These models do not eliminate non-proportionality; their hazard ratios represent average associations over the respective intervals rather than constant or time-specific effects.

In a further sensitivity analysis, recipients were dichotomized into low- and high-risk groups based on the sex-specific median cutoff of the best-performing index, and graft survival within 3 years was compared using Kaplan–Meier estimates with the log-rank test. A data-derived, outcome-optimized threshold was deliberately avoided to prevent cutpoint-optimization bias.

Finally, to assess whether TBMMI contributed prognostic information beyond routine clinical assessment, we fitted a Cox proportional hazards model for graft failure containing variables available at pre-transplant evaluation—age, sex, BMI, and MELD-Na—and compared it with the same model extended by either SMI or TBMMI. Nested models were compared by likelihood-ratio test, and discrimination was quantified with Harrell’s C-index; differences in C-index between correlated models were tested using the method for two concordance estimates from the same data.

All statistical analyses were performed using R software (version 4.5.1; R Foundation for Statistical Computing, Vienna, Austria). A two-sided *p* value of less than 0.05 was considered statistically significant.

## 3. Results

### 3.1. Baseline Characteristics

A total of 802 adult LDLT recipients (508 males, 294 females; median age 58.0 years) were included in the analysis (Table 1). Cr was measured the day before transplantation in 746 recipients, on the day of transplantation in 45, and two days before in 11; CysC was measured on the day of transplantation in 799 and the day before in 3. The preoperative CT was performed a median of 2 days before transplantation (interquartile range, 2–7 days; range, 1–45 days). Consequently, the interval between the CT examination and the Cr measurement was a median of 1 day (interquartile range, 1–6 days; maximum, 44 days).

The median MELD-Na score was 11 (interquartile range, 8–17). The predominant etiologies of liver disease were hepatitis B virus infection (45.9%) and alcohol-related liver disease (33.3%). CT-defined sarcopenia was present in 184 recipients (22.9%). Compared with the patients without CT-defined sarcopenia, the sarcopenia group had a higher proportion of male recipients (81.0% vs. 58.1%) and a lower BMI (median 21.2 vs. 24.1), along with significantly higher MELD-Na scores (median 15.5 vs. 11.0) and greater frequencies of massive transfusion (50.5% vs. 26.1%), as well as a higher proportion of alcohol-related liver disease (42.4% vs. 30.6%). Muscle mass indices (SMI, TBMMI, ASMI) and CCR were also significantly lower in the sarcopenia group (all *p* < 0.001), whereas pSMI did not differ significantly between the groups (*p* = 0.159).

Compared with the 802 analyzed recipients, the 104 excluded recipients had greater baseline disease severity and comorbidity burden (MELD-Na 20.0 [15.0–24.5] vs. 11.0 [8.0–17.0]; hypertension 51.0% vs. 25.1%; intractable ascites 65.4% vs. 47.6%; all *p* ≤ 0.001), more frequently required intraoperative massive transfusion (53.8% vs. 31.7%; *p* < 0.001), and had worse outcomes (graft failure 17.3% vs. 6.2%; mortality 16.3% vs. 5.5%; both *p* < 0.001) (Supplementary Table S1).

### 3.2. Serum Indices and Identification of CT-Defined Sarcopenia

**Figure 2:**
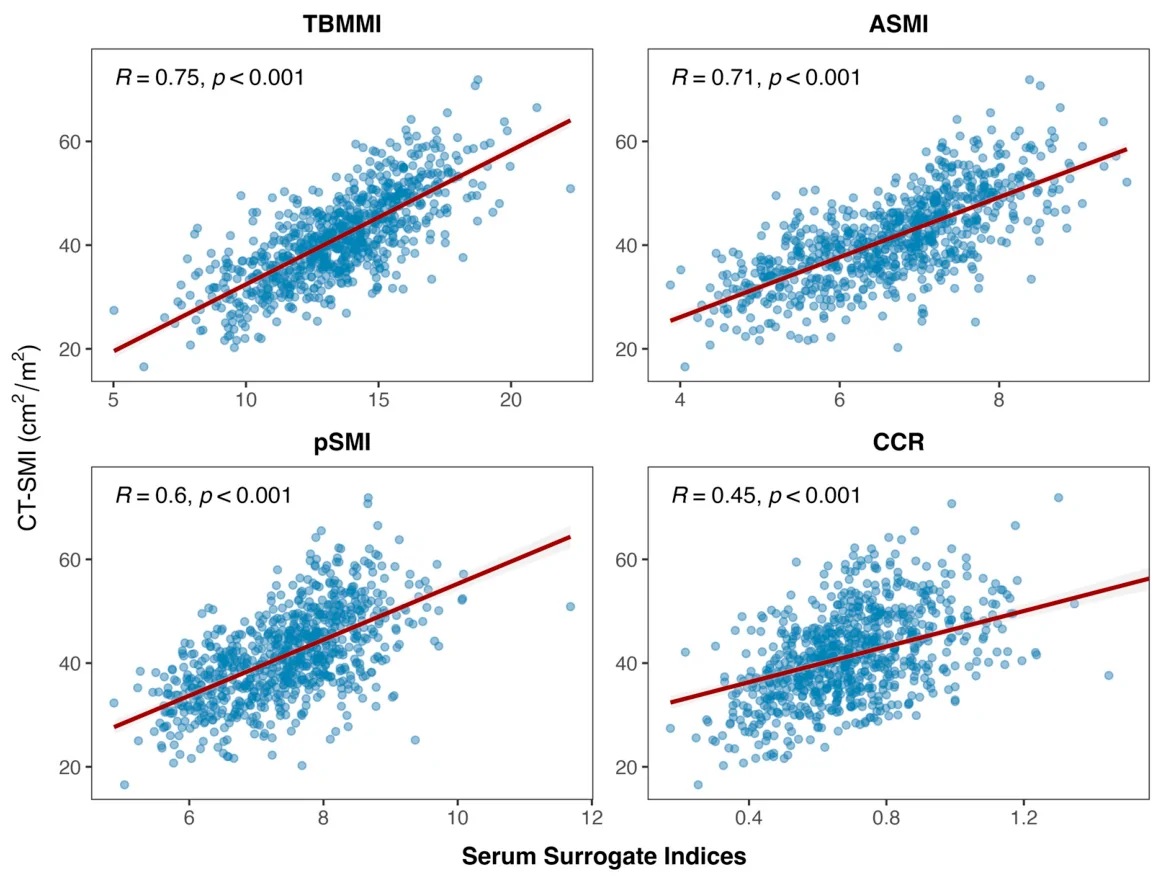
Correlation between serum surrogate indices and CT-derived skeletal muscle index. Each blue circle represents an individual LDLT recipient and the red line is the least-squares linear regression line. Abbreviations: ASMI, appendicular skeletal muscle mass index; CCR, creatinine-to-cystatin C ratio; CT-SMI, computed tomography-derived skeletal muscle index; pSMI, predicted skeletal muscle index; R, Pearson correlation coefficient; TBMMI, total body muscle mass index.

Among the serum surrogates, TBMMI exhibited the strongest correlation with SMI (Pearson *r* = 0.75), compared with ASMI (*r* = 0.71), pSMI (*r* = 0.60), and CCR (*r* = 0.45) (Figure 2). TBMMI showed the strongest correlation with SMI in both males (Pearson *r* = 0.713) and females (Pearson *r* = 0.545) (Supplementary Table S2).

**Figure 3:**
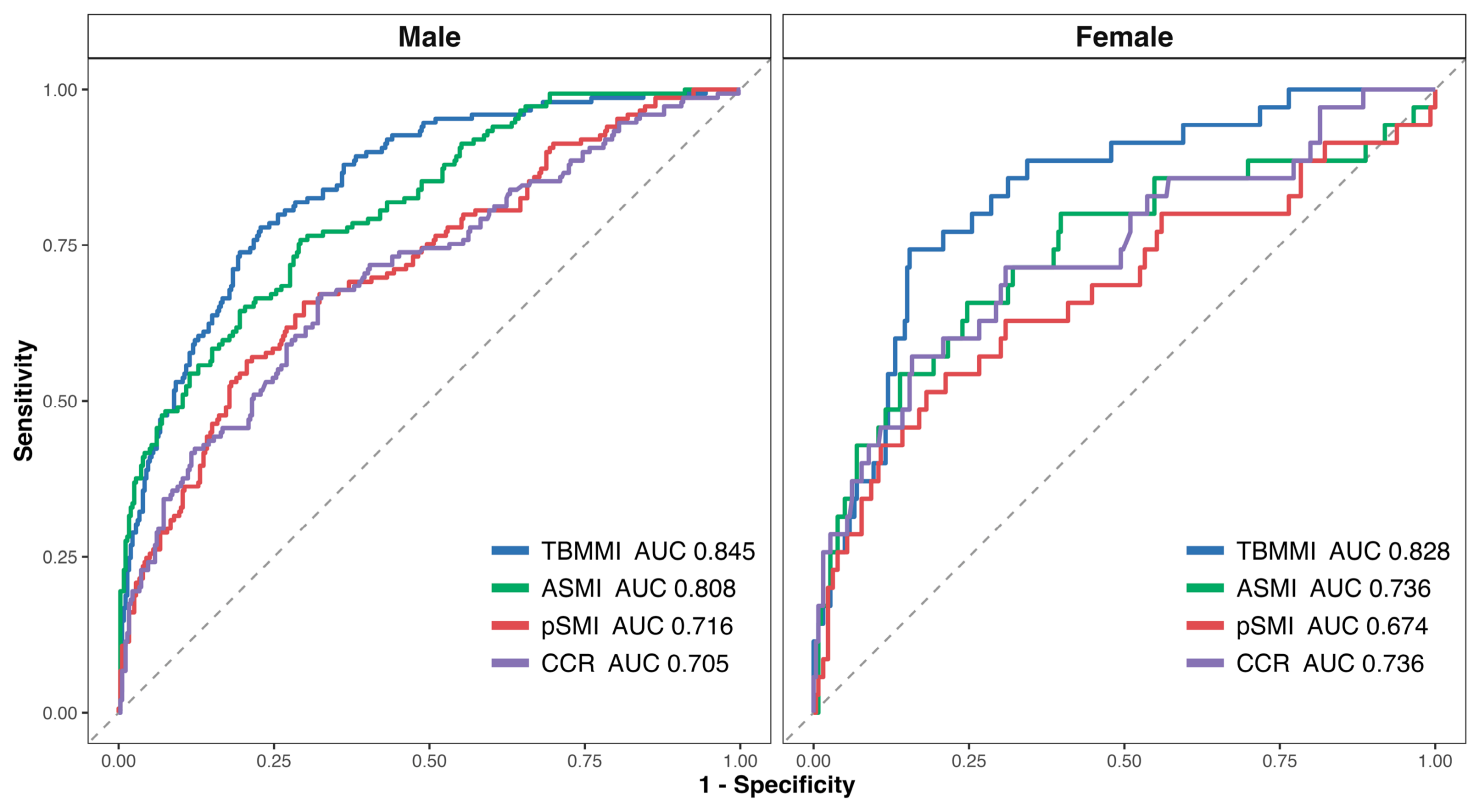
Sex-stratified receiver operating characteristic (ROC) curves of serum surrogate indices for detecting CT-defined sarcopenia. Abbreviations: ASMI, appendicular skeletal muscle mass index; AUC, area under the receiver operating characteristic curve; CCR, creatinine-to-cystatin C ratio; pSMI, predicted skeletal muscle index; TBMMI, total body muscle mass index.

For the discrimination of CT-defined sarcopenia, TBMMI demonstrated the highest AUC in both males (0.845; 95% CI, 0.808–0.881) and females (0.828; 95% CI, 0.758–0.898), outperforming ASMI (0.808 in males, 0.736 in females), pSMI (0.716 in males, 0.674 in females), and CCR (0.705 in males, 0.736 in females) (Figure 3).

In DeLong testing, the AUC for TBMMI was significantly higher than that for pSMI in both sexes (*p* < 0.001 in males, *p* = 0.005 in females) and than that for CCR in both sexes (*p* < 0.001 in males, *p* = 0.010 in females). Additionally, it was higher than that for ASMI in males (*p* = 0.021), with a similar but non-significant trend observed in females (*p* = 0.051) (Table 2). The Youden-derived cutoffs for TBMMI were 14.02 kg/m^2^ in males (sensitivity 78%, specificity 77%) and 10.06 kg/m^2^ in females (sensitivity 74%, specificity 85%) (Table 2). Internal validation using bootstrap resampling (1000 resamples) showed negligible differences between apparent and optimism-corrected AUCs for TBMMI (males: 0.845 vs. 0.844; females: 0.828 vs. 0.828), suggesting limited optimism in the apparent diagnostic performance. Optimism was larger for the Youden index, whose cutoff was re-estimated in each resample (males: 0.550 vs. 0.526; females: 0.588 vs. 0.546), suggesting that the proposed sex-specific cutoffs warrant external validation (Supplementary Table S3).

TBMMI alone explained more of the variance in SMI (*R*^2^ = 0.559) than the anthropometric model containing age, sex, and BMI (0.442), and adding TBMMI to that model raised the explained variance to 0.584 (Δ*R*^2^ = 0.142; *p* < 0.001); the increments were smaller for ASMI (Δ*R*^2^ = 0.078) and CCR (Δ*R*^2^ = 0.069) and absent for pSMI (Δ*R*^2^ < 0.001, *p* = 0.81). Discrimination of CT-defined sarcopenia showed the same ordering: adding TBMMI to age and BMI raised the AUC from 0.789 to 0.851 in men (ΔAUC = 0.062, *p* < 0.001) and from 0.727 to 0.829 in women (ΔAUC = 0.103, *p* = 0.07), whereas pSMI added nothing in either sex (Supplementary Table S4).

### 3.3. TBMMI and Post-Transplant Outcomes

Median follow-up estimated by the reverse Kaplan–Meier method was 679 days (95% CI, 627–741) for graft failure. Within the 3-year analysis window, 50 graft failures and 44 deaths occurred. The numbers at risk were 672, 559, and 107 at 180 days, 1 year, and 3 years for graft failure and 676, 563, and 108 for all-cause mortality (Supplementary Table S5).

For the primary outcome of graft failure, each 1 kg/m^2^ decrease in TBMMI was independently associated with graft failure, with adjusted hazard ratios of 1.42 (95% CI, 1.14–1.78; *p* = 0.002), 1.28 (95% CI, 1.05–1.56; *p* = 0.016), and 1.20 (95% CI, 1.02–1.40; *p* = 0.024) when follow-up was truncated at 180 days, 1 year, and 3 years, respectively (Table 3). The average association was larger over shorter follow-up windows, although these estimates should not be interpreted as constant effects within each interval. In particular, the 180-day estimate was based on only 24 graft-failure events and should be interpreted cautiously. Regarding all-cause mortality, each 1 kg/m^2^ decrease in TBMMI was independently associated with an increased risk of death, with adjusted hazard ratios of 1.40 (95% CI, 1.09–1.81; *p* = 0.010), 1.27 (95% CI, 1.01–1.59; *p* = 0.042), and 1.18 (95% CI, 1.00–1.40; *p* = 0.048) when follow-up was truncated at 180 days, 1 year, and 3 years, respectively (Supplementary Table S6).

The association between lower TBMMI and graft failure was not significantly modified by renal function (adjusted HR, 1.10 for eGFR < 60 and 1.20 for eGFR ≥ 60 mL/min/1.73 m^2^; *p* for interaction = 0.743) or MELD-Na severity (adjusted HR, 1.09 for MELD-Na < 15 and 1.25 for MELD-Na ≥ 15; *p* for interaction = 0.265) (Supplementary Table S7). The estimate in the impaired renal-function subgroup should be interpreted with caution, as it comprised only 46 recipients with 3 graft failures.

TBMMI, recipient age, and the graft-to-recipient weight ratio were adequately represented by linear terms for both outcomes, whereas the MELD-Na score departed from linearity; modeling it as a natural cubic spline left the TBMMI estimates essentially unchanged (HR, 1.20; 95% CI, 1.03–1.41 for graft failure and HR, 1.20; 95% CI, 1.01–1.42 for mortality). The proportional hazards assumption was not satisfied for TBMMI in either outcome (Schoenfeld test, *p* = 0.010 for graft failure and *p* = 0.038 for all-cause mortality); the hazard ratio for the interaction between TBMMI and log(time) was 1.07 (*p* = 0.081) for graft failure (Supplementary Table S8).

**Figure 4:**
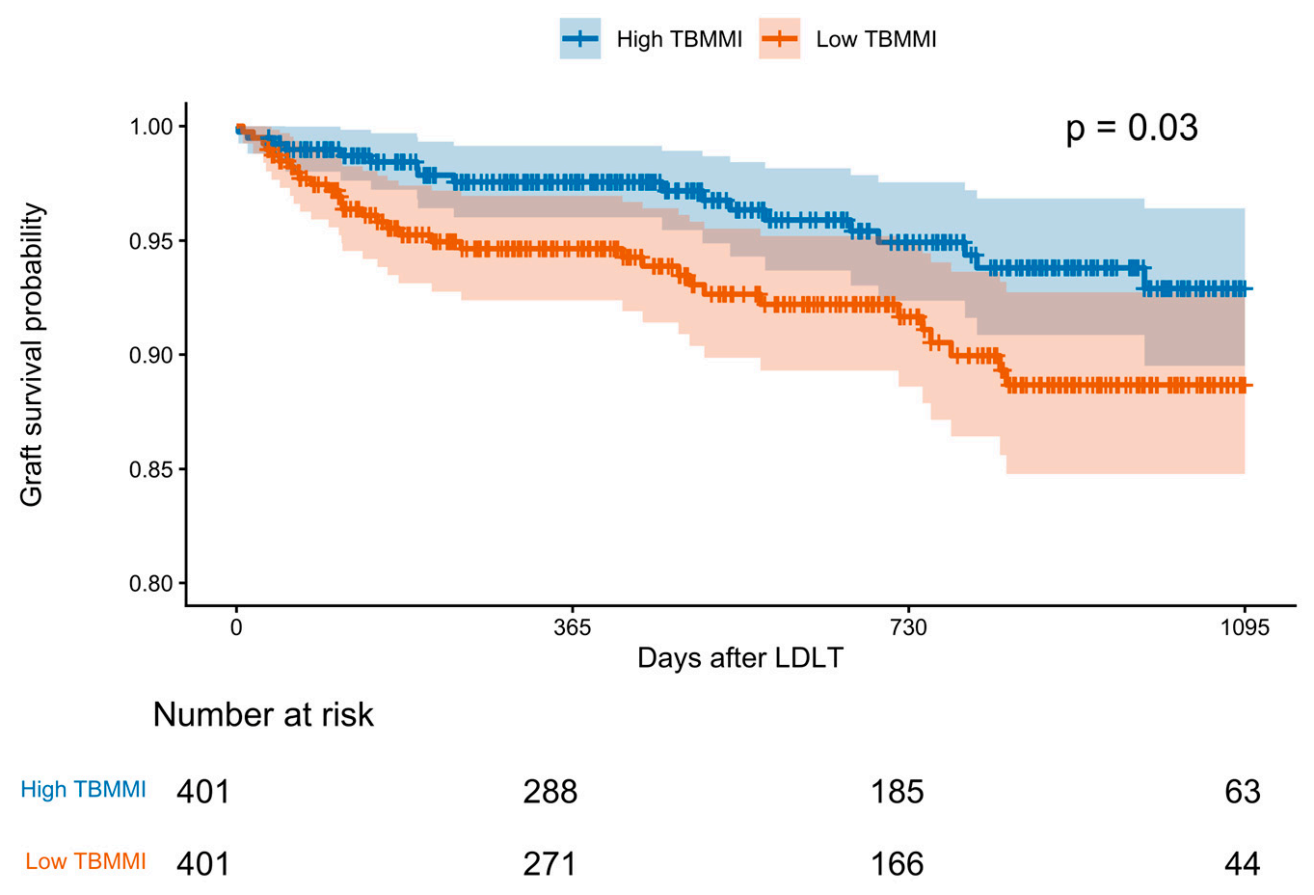
Kaplan–Meier survival analysis of 3-year graft failure according to TBMMI category. Recipients were dichotomized at the sex-specific median TBMMI (14.55 kg/m^2^ for males and 11.58 kg/m^2^ for females). Shaded areas represent 95% confidence intervals. The grey areas indicate where the two semi-transparent confidence bands overlap. Vertical tick marks denote censored observations. The *x*-axis is marked at 1-year intervals. Abbreviations: LDLT, living donor liver transplantation; TBMMI, total body muscle mass index.

In the sensitivity analysis, recipients were dichotomized based on the sex-specific median TBMMI values, which were 14.55 kg/m^2^ for males and 11.58 kg/m^2^ for females. Within the first 3 years, the low-TBMMI group exhibited significantly worse graft survival compared with the high-TBMMI group, as demonstrated by Kaplan–Meier analysis (log-rank *p* = 0.030). Graft survival rates were 95.2% vs. 98.5% at 180 days, 94.6% vs. 97.6% at 1 year, and 88.7% vs. 92.9% at 3 years (Figure 4).

The base model containing age, sex, BMI, and MELD-Na yielded a C-index of 0.657 for 3-year graft failure. Adding TBMMI improved model fit (likelihood-ratio *p* = 0.009), as did adding SMI (*p* = 0.003). The corresponding gains in discrimination were modest and did not reach statistical significance for either index (Δ*C*-index +0.024, *p* = 0.238 for TBMMI and +0.019, *p* = 0.423 for SMI), and the two extended models did not differ significantly from each other (Δ*C*-index +0.004, *p* = 0.84) (Supplementary Table S9).

## 4. Discussion

In this single-center retrospective study of 802 adult LDLT recipients, we evaluated serum creatinine- and cystatin C-based muscle indices. Among the four serum surrogate indices examined (TBMMI, ASMI, pSMI, and CCR), TBMMI showed the strongest correlation with SMI and the highest accuracy in identifying CT-defined sarcopenia. Furthermore, TBMMI was independently associated with graft failure and mortality following LDLT. To our knowledge, this is one of the first studies to link a CT-validated serum muscle index with post-transplant clinical outcomes in an LDLT cohort.

The prevalence of CT-defined sarcopenia in our study was 22.9% (184/802). According to a recent meta-analysis of liver transplant recipients, the pooled prevalence of sarcopenia was 40.7% (95% CI, 32.1–49.6; 25 studies) and 38.8% (95% CI, 23.3–55.5; 10 studies) in the Asian subgroup [28]. The wide confidence intervals reflect considerable heterogeneity among studies, largely driven by differences in muscle measurement modalities and variations in recipient populations across different eras and countries. Notably, our estimate aligns most closely with the approximately 21% reported in a Japanese LDLT cohort that, like ours, defined sarcopenia by L3-SMI using sex-specific cutoffs derived from a healthy living-donor population [29].

Serum-based indices are increasingly being investigated as accessible surrogates for muscle mass in patients with liver disease. Koizumi et al. reported that the CCR discriminated CT-defined sarcopenia in patients with hepatitis C-related cirrhosis after sustained virologic response, and that higher CCR values were associated with better overall survival [23]. Hwang et al. found that TBMM discriminated low SMI, as determined by BIA, more accurately than CCR in patients with metabolic dysfunction-associated steatotic liver disease; however, this was a cross-sectional screening study that did not assess clinical outcomes [24]. Similarly, Ichikawa et al. reported that in patients with liver disease, TBMM correlated much more strongly with CT-measured skeletal muscle than CCR alone (*r* = 0.804 vs. 0.293), although that study also did not evaluate clinical outcomes [30]. Although direct comparisons across cohorts are limited by differences in population characteristics and sarcopenia criteria, within our own cohort, CCR showed lower discrimination (AUC 0.705 in males, 0.736 in females) than TBMMI (AUC 0.845 in males and 0.828 in females; DeLong *p* < 0.001 and *p* = 0.010, respectively), indicating that TBMMI reflects muscle mass more accurately than CCR alone.

In a waiting-list cohort, Mauro et al. developed the Sarcopenia HIBA score using sex, BMI, Child–Pugh class, and CCR to identify CT-defined sarcopenia and predict waiting-list mortality [17]. Because the model incorporates the Child–Pugh class, a marker of liver disease severity, the resulting score inevitably reflects hepatic dysfunction in addition to muscle depletion. To focus more directly on muscle mass, we sought an objective index derived from routine laboratory and anthropometric measures that correlates directly with SMI. TBMMI may thus serve both as a practical tool for pre-transplant sarcopenia assessment and as a marker independently associated with post-transplant graft failure and mortality.

As the transplant population ages, TBMMI may play an increasingly important role in perioperative patient assessment. According to the United Network for Organ Sharing database, both the overall number and proportion of liver transplants in older recipients (≥65 years) have increased over time, with the proportion of older recipients exceeding 20% after 2016 [31]. Current EASL guidelines emphasize that LT candidacy in older recipients should be evaluated based on physiological reserve—such as frailty and sarcopenia—rather than chronological age alone [14]. TBMMI provides a reproducible and easily calculated method for such assessment, using routinely collected data. Thus, TBMMI may offer an accessible approach to risk assessment as the need for objective evaluation of physiological reserve in older LT candidates continues to grow.

This study has several limitations. First, it was a single-center, retrospective analysis of an Asian LDLT cohort; therefore, external validation in deceased-donor, Western, and multiethnic cohorts is necessary before broader application. Second, the original TBMM equation was derived from a healthy, community-dwelling population [25], and creatinine and cystatin C may be affected by renal and hepatic dysfunction. Although TBMMI was validated against SMI in our LDLT cohort and subgroup analyses showed no significant effect modification by eGFR or MELD-Na, its validity in patients with advanced renal or hepatic dysfunction requires further investigation. Third, although TBMMI demonstrated the strongest correlation with SMI across both sexes, the estimates in women were less precise than in men. The female stratum contained only 35 sarcopenia cases, resulting in wider confidence intervals for both the correlation coefficient and the AUC; consequently, these results should be interpreted with caution. Fourth, the proportional hazards assumption was not satisfied for TBMMI, and the truncated Cox models provide average hazard ratios over the specified follow-up windows rather than time-specific effects. Moreover, the limited number of early events resulted in imprecise 180-day estimates. Fifth, while TBMMI correlates well with SMI, both indices primarily measure muscle quantity. Given the growing consensus that muscle quality and functional metrics (e.g., grip strength and physical performance) are integral to the clinical impact of sarcopenia [1,2], the potential prognostic role of these factors remains an important area for future investigation.

## 5. Conclusions

In this cohort of 802 adult LDLT recipients, TBMMI showed the strongest correlation with CT-derived SMI and identified CT-defined sarcopenia with good accuracy in both sexes, outperforming pSMI and CCR. In addition, lower TBMMI was independently associated with graft failure and postoperative mortality, extending previous validation studies by demonstrating its prognostic relevance after LDLT. Further studies should externally validate TBMMI, establish outcome-based thresholds, and assess whether serial measurements and functional measures improve risk stratification.

## Figures and Tables

**Table 1:** Baseline characteristics by CT-defined sarcopenia.

Variables	Overall (*N* = 802)	No Sarcopenia (*N* = 618)	Sarcopenia (*N* = 184)	*p* Value
Age, years	58.0 [52.0–64.0]	59.0 [53.0–64.0]	57.0 [50.0–64.0]	0.114
Male sex	508 (63.3)	359 (58.1)	149 (81.0)	<0.001
BMI, kg/m^2^	23.5 [21.3–25.8]	24.1 [22.1–26.4]	21.2 [19.4–23.4]	<0.001
MELD-Na score	11.0 [8.0–17.0]	11.0 [8.0–15.0]	15.5 [10.0–22.0]	<0.001
Etiologies				
HBV	368 (45.9)	290 (46.9)	78 (42.4)	0.318
HCV	31 (3.9)	22 (3.6)	9 (4.9)	0.545
Alcohol	267 (33.3)	189 (30.6)	78 (42.4)	0.004
Biliary	55 (6.9)	43 (7.0)	12 (6.5)	0.969
Other etiology	114 (14.2)	96 (15.5)	18 (9.8)	0.066
Diabetes	248 (30.9)	191 (30.9)	57 (31.0)	1.000
Hypertension	201 (25.1)	160 (25.9)	41 (22.3)	0.371
GRWR	1.1 [0.9–1.3]	1.1 [0.9–1.3]	1.1 [0.9–1.3]	0.194
Donor age, years	32.0 [27.0–40.0]	32.0 [26.0–39.0]	34.0 [27.0–41.0]	0.133
Donor male sex	490 (61.1)	396 (64.1)	94 (51.1)	0.002
Massive transfusion	254 (31.7)	161 (26.1)	93 (50.5)	<0.001
SMI, cm^2^/m^2^	40.9 [35.4–46.9]	43.7 [38.4–49.1]	34.9 [28.5–37.6]	<0.001
TBMMI, kg/m^2^	13.6 [11.8–15.2]	13.9 [12.1–15.5]	12.6 [10.9–13.7]	<0.001
ASMI, kg/m^2^	6.7 [5.9–7.3]	6.9 [5.9–7.5]	6.5 [5.9–7.0]	<0.001
pSMI, kg/m^2^	7.5 [6.7–8.1]	7.6 [6.6–8.1]	7.4 [6.9–7.9]	0.159
CCR	0.7 [0.6–0.8]	0.7 [0.6–0.8]	0.6 [0.5–0.7]	<0.001

Sarcopenia was defined as an L3 skeletal muscle index below 39.9 cm^2^/m^2^ in men and below 28.9 cm^2^/m^2^ in women. Normality was assessed with the Shapiro–Wilk test; all continuous variables were non-normally distributed and are presented as median [interquartile range] and compared with the Mann–Whitney U test. Categorical variables are presented as number (%) and compared with the chi-square test or Fisher exact test as appropriate. Etiologies are not mutually exclusive; some recipients had more than one etiology. Abbreviations: ASMI, appendicular skeletal muscle mass index; BMI, body mass index; CCR, creatinine-to-cystatin C ratio; GRWR, graft-to-recipient weight ratio; HBV, hepatitis B virus; HCV, hepatitis C virus; MELD-Na, Model for End-Stage Liver Disease–Sodium; pSMI, predicted skeletal muscle index; SMI, skeletal muscle index; TBMMI, total body muscle mass index.

**Table 2:** Sex-stratified diagnostic performance of serum surrogate indices for detecting CT-defined sarcopenia.

Sex	Index	AUC (95% CI)	Cutoff	Sensitivity	Specificity	*p* Value
Male	TBMMI	0.845 (0.808–0.881)	14.02	0.78	0.77	Ref.
Male	ASMI	0.808 (0.767–0.849)	7.06	0.76	0.71	0.021
Male	pSMI	0.716 (0.666–0.766)	7.75	0.66	0.70	<0.001
Male	CCR	0.705 (0.653–0.756)	0.70	0.67	0.67	<0.001
Female	TBMMI	0.828 (0.758–0.898)	10.06	0.74	0.85	Ref.
Female	ASMI	0.736 (0.632–0.840)	5.22	0.66	0.75	0.051
Female	pSMI	0.674 (0.563–0.786)	6.03	0.51	0.82	0.005
Female	CCR	0.736 (0.638–0.835)	0.45	0.57	0.84	0.010

*p* values are from DeLong tests comparing each index against TBMMI (reference) within each sex. CT-defined sarcopenia was present in 149 of 508 male recipients (29.3%), and in 35 of 294 female recipients (11.9%). Cutoffs are expressed in kg/m^2^ for TBMMI, ASMI, and pSMI and are dimensionless for CCR. Abbreviations: ASMI, appendicular skeletal muscle mass index; AUC, area under the receiver operating characteristic curve; CCR, creatinine-to-cystatin C ratio; CI, confidence interval; pSMI, predicted skeletal muscle index; TBMMI, total body muscle mass index.

**Table 3:** Univariable and prespecified multivariable Cox models for graft failure.

	Events/N	Univariable		Prespecified Multivariable	
		HR (95% CI)	*p* Value	HR (95% CI)	*p* Value
Panel A. Cox Regression Analysis for 3-Year Graft Failure					
TBMMI (per 1 kg/m^2^ lower)	50/802	1.18 (1.06, 1.32)	0.003	1.20 (1.02, 1.40)	0.024
Recipient age (per year)		1.02 (0.99, 1.05)	0.3	1.03 (1.00, 1.07)	0.058
Recipient sex, male		0.62 (0.35, 1.08)	0.089	0.90 (0.46, 1.76)	0.8
MELD-Na score (per point)		1.05 (1.02, 1.09)	0.006	1.04 (0.99, 1.09)	0.12
Graft-to-recipient weight ratio		0.93 (0.37, 2.33)	0.9	0.48 (0.17, 1.34)	0.2
Massive transfusion		1.80 (1.03, 3.15)	0.039	1.25 (0.67, 2.31)	0.5
Panel B. TBMMI by length of follow-up					
Graft failure, 180 days	24/802	1.34 (1.14, 1.57)	<0.001	1.42 (1.14, 1.78)	0.002
Graft failure, 1 year	29/802	1.31 (1.13, 1.52)	<0.001	1.28 (1.05, 1.56)	0.016

Hazard ratios are expressed per 1 kg/m^2^ lower TBMMI, with TBMMI modeled as a continuous variable. The multivariable model is the prespecified model containing TBMMI, recipient age, recipient sex, the MELD-Na score, the graft-to-recipient weight ratio, and massive transfusion; the same six covariates were fitted in every multivariable model. Abbreviations: CI, confidence interval; HR, hazard ratio; MELD-Na, Model for End-Stage Liver Disease–Sodium; TBMMI, total body muscle mass index.

## Data Availability

The dataset used and/or analyzed during the current study is available from the corresponding author (Jun-Gol Song, jungol.song@amc.seoul.kr) upon reasonable request.

## Supplementary Materials

The following supporting information can be downloaded at https://www.mdpi.com/article/10.3390/jcm15176889/s1, Table S1: Baseline characteristics and clinical outcomes of included versus excluded recipients; Table S2: Sex-stratified Pearson correlation between the creatinine- and cystatin C-based muscle indices and the CT-derived skeletal muscle index; Table S3: Bootstrap-corrected optimism of diagnostic performance for TBMMI; Table S4: Contribution of the serum muscle indices beyond anthropometric reference models; Table S5: Follow-up structure, number at risk, and cumulative events; Table S6: Sensitivity of the TBMMI–outcome association to the multivariable modeling strategy; Table S7: Association between TBMMI and graft failure according to renal function and liver disease severity; Table S8: Assessment of the proportional hazards assumption and of the functional form of continuous covariates; Table S9: Incremental prognostic value of TBMMI and SMI for graft failure.

## Abbreviations

The following abbreviations are used in this manuscript:

AIC	Akaike information criterion
ASM	appendicular skeletal muscle mass
ASMI	appendicular skeletal muscle mass index
AUC	area under the receiver operating characteristic curve
BIA	bioelectrical impedance analysis
BMI	body mass index
BW	body weight
CCR	creatinine-to-cystatin C ratio
CI	confidence interval
Cr	creatinine
CT	computed tomography
CysC	cystatin C
eGFR	estimated glomerular filtration rate
GRWR	graft-to-recipient weight ratio
Hb	hemoglobin
HBV	hepatitis B virus
HCV	hepatitis C virus
HR	hazard ratio
HU	Hounsfield units
L3	third lumbar vertebra
LDLT	living donor liver transplantation
LT	liver transplantation
MELD	Model for End-Stage Liver Disease
MELD-Na	Model for End-Stage Liver Disease–Sodium
pSMI	predicted skeletal muscle index
ROC	receiver operating characteristic
SMI	skeletal muscle index
TBMM	total body muscle mass
TBMMI	total body muscle mass index
