# Supplementary material for: Serum Creatinine/Cystatin C-Based Muscle Indices for Sarcopenia and Graft Failure After Living Donor Liver Transplantation
Source: J Clin Med. 2026 Sep 5;15(17):6889. doi: 10.3390/jcm15176889 (PMC13566252; doi:10.3390/jcm15176889)
Supplement: Supplementary file 1 [file jcm-15-06889-s001.zip › jcm-4500007-supplementary.pdf]

**Table S1.** Baseline characteristics and clinical outcomes of included versus excluded recipients.

| Characteristic | Included ($n$ = 802) | Excluded ($n$ = 104) | $p$ |
|---|---|---|---|
| Age, years | 58.0 [52.0–64.0] | 58.0 [54.0–65.0] | 0.314 |
| Male sex | 508 (63.3) | 68 (65.4) | 0.765 |
| Body mass index, kg/m² | 23.5 [21.3–25.8] | 23.4 [20.4–25.6] | 0.349 |
| MELD-Na | 11.0 [8.0–17.0] | 20.0 [15.0–24.5] | <0.001 |
| *Etiology of liver disease* | | | |
| Hepatitis B | 368 (45.9) | 34 (32.7) | 0.015 |
| Hepatitis C | 31 (3.9) | 3 (2.9) | 0.825 |
| Alcohol | 267 (33.3) | 47 (45.2) | 0.022 |
| Biliary | 55 (6.9) | 1 (1.0) | 0.033 |
| Other | 114 (14.2) | 25 (24.0) | 0.019 |
| Diabetes mellitus | 248 (30.9) | 41 (39.4) | 0.101 |
| Hypertension | 201 (25.1) | 53 (51.0) | <0.001 |
| Intractable ascites | 382 (47.6) | 68 (65.4) | 0.001 |
| Graft-to-recipient weight ratio | 1.1 [0.9–1.3] | 1.1 [1.0–1.3] | 0.164 |
| Donor age, years | 32.0 [27.0–40.0] | 32.0 [26.0–41.0] | 0.988 |
| Donor male sex | 490 (61.1) | 64 (61.5) | 1.000 |
| Massive transfusion | 254 (31.7) | 56 (53.8) | <0.001 |
| Graft failure | 50 (6.2) | 18 (17.3) | <0.001 |
| Death | 44 (5.5) | 17 (16.3) | <0.001 |

Normality was assessed with the Shapiro–Wilk test; all continuous variables were non-normally distributed and are presented as median [interquartile range] and compared with the Mann–Whitney U test. Categorical variables are presented as number (%) and compared with the chi-square test or Fisher exact test as appropriate. Graft failure and death were counted with follow-up administratively truncated at 3 years. Abbreviations: MELD-Na, Model for End-Stage Liver Disease–Sodium.

**Table S2.** Sex-stratified Pearson correlation between the creatinine– and cystatin C–based muscle indices and the CT-derived skeletal muscle index.

| | Male | | Female | |
|---|---|---|---|---|
| Index | $r$ (95% CI) | $p$ | $r$ (95% CI) | $p$ |
| TBMMI | 0.713 (0.668, 0.754) | <0.001 | 0.545 (0.460, 0.621) | <0.001 |
| ASMI | 0.659 (0.607, 0.706) | <0.001 | 0.496 (0.404, 0.577) | <0.001 |
| pSMI | 0.464 (0.392, 0.529) | <0.001 | 0.360 (0.256, 0.456) | <0.001 |
| CCR | 0.350 (0.271, 0.424) | <0.001 | 0.259 (0.149, 0.363) | <0.001 |

Pearson correlation coefficients with 95% confidence intervals obtained by Fisher z transformation, computed separately in men and women. Abbreviations: ASMI, appendicular skeletal muscle mass index; CCR, creatinine-to-cystatin C ratio; CI, confidence interval; CT, computed tomography; pSMI, predicted skeletal muscle index; TBMMI, total body muscle mass index.

**Table S3.** Bootstrap-corrected optimism of diagnostic performance for TBMMI.

| Marker | Sex | AUC (apparent) | AUC (corrected) | Youden J (apparent) | Youden J (corrected) |
|---|---|---|---|---|---|
| TBMMI | Male | 0.845 | 0.844 | 0.550 | 0.526 |
| TBMMI | Female | 0.828 | 0.828 | 0.588 | 0.546 |

Apparent estimates were obtained in the original cohort; corrected estimates were obtained by subtracting the optimism estimated from 1,000 bootstrap resamples, in which the Youden-derived cutoff was re-estimated in each resample and applied back to the original data. Abbreviations: AUC, area under the receiver operating characteristic curve; J, Youden index; TBMMI, total body muscle mass index.

**Table S4.** Contribution of the serum muscle indices beyond anthropometric reference models.

| Model | Estimate (95% CI) | Increment | *p* |
|---|---|---|---|
| *(a) Variance in SMI explained ($R^2$)* | | | |
| Body mass index alone | 0.246 | — | — |
| Age, sex, body mass index (reference) | 0.442 | — | — |
| TBMMI alone | 0.559 | — | — |
| ASMI alone | 0.500 | — | — |
| pSMI alone | 0.359 | — | — |
| CCR alone | 0.198 | — | — |
| Reference + TBMMI | 0.584 | +0.142 | <0.001 |
| Reference + ASMI | 0.520 | +0.078 | <0.001 |
| Reference + CCR | 0.511 | +0.069 | <0.001 |
| Reference + pSMI | 0.442 | 0.000 | 0.81 |
| *(b) Discrimination of CT-defined sarcopenia (area under the curve)* | | | |
| Men: age, body mass index (reference) | 0.789 (0.746, 0.832) | — | — |
| Men: reference + TBMMI | 0.851 (0.815, 0.886) | +0.062 | <0.001 |
| Men: reference + CCR | 0.833 (0.796, 0.870) | +0.044 | <0.001 |
| Men: reference + ASMI | 0.819 (0.780, 0.858) | +0.031 | 0.01 |
| Men: reference + pSMI | 0.789 (0.746, 0.832) | +0.001 | 0.73 |
| Women: age, body mass index (reference) | 0.727 (0.620, 0.833) | — | — |
| Women: reference + TBMMI | 0.829 (0.758, 0.901) | +0.103 | 0.07 |
| Women: reference + CCR | 0.773 (0.687, 0.859) | +0.047 | 0.35 |
| Women: reference + ASMI | 0.766 (0.684, 0.847) | +0.039 | 0.41 |
| Women: reference + pSMI | 0.726 (0.620, 0.833) | 0.000 | 0.94 |

All analyses were performed in the 802 recipients. In panel (a) the increment is the change in $R^2$ relative to the reference model containing age, sex, and body mass index, and the *p* value is from the incremental F test; adding pSMI changed $R^2$ from 0.44210 to 0.44214 ($\Delta R^2 = 0.00004$). In panel (b) all models are fitted within sex because the CT thresholds are sex-specific, the increment is the change in the area under the curve relative to the reference model containing age and body mass index, and the *p* value is from the DeLong test.

CT-defined sarcopenia was present in 149 of 508 men and 35 of 294 women.

Abbreviations: ASMI, appendicular skeletal muscle mass index; CCR, creatinine-to-cystatin C ratio; CI, confidence interval; CT, computed tomography; pSMI, predicted skeletal muscle index; SMI, skeletal muscle index; TBMMI, total body muscle mass index.

**Table S5.** Follow-up structure, number at risk, and cumulative events.

| Outcome | Time since transplantation (days) | Number at risk | Cumulative events | Kaplan–Meier estimate, % (95% CI) |
|---|---|---|---|---|
| Graft failure | 180 | 672 | 24 | 96.9 (95.6–98.1) |
| Graft failure | 365 | 559 | 29 | 96.1 (94.7–97.5) |
| Graft failure | 1095 | 107 | 50 | 90.8 (88.2–93.5) |
| Mortality | 180 | 676 | 19 | 97.5 (96.4–98.6) |
| Mortality | 365 | 563 | 23 | 96.9 (95.6–98.2) |
| Mortality | 1095 | 108 | 44 | 91.6 (89.0–94.2) |

Graft failure was defined as recipient death or re-transplantation. Estimates are Kaplan–Meier survival probabilities (that is, freedom from the event) with 95% confidence intervals; the number at risk is the number of recipients remaining under follow-up at each time point. Abbreviations: CI, confidence interval.

**Table S6.** Sensitivity of the TBMMI–outcome association to the multivariable modeling strategy.

| | | Prespecified model | | Extended model | | Backward elimination | | |
|---|---|---|---|---|---|---|---|---|
| Outcome and follow-up | Events | HR (95% CI) | *p* | HR (95% CI) | *p* | HR (95% CI) | *p* | Covariates retained by backward elimination |
| Graft failure, ≤180 days | 24 | 1.42 (1.14, 1.78) | 0.002 | 1.42 (1.14, 1.76) | 0.002 | 1.45 (1.20, 1.76) | <0.001 | TBMMI, age, MELD-Na, GRWR |
| Graft failure, ≤1 year | 29 | 1.28 (1.05, 1.56) | 0.016 | 1.28 (1.05, 1.56) | 0.013 | 1.35 (1.13, 1.60) | <0.001 | TBMMI, age, MELD-Na, GRWR |
| Graft failure, ≤3 years | 50 | 1.20 (1.02, 1.40) | 0.024 | 1.20 (1.02, 1.40) | 0.024 | 1.22 (1.06, 1.39) | 0.004 | TBMMI, age, MELD-Na, GRWR |
| Mortality, ≤180 days | 19 | 1.40 (1.09, 1.81) | 0.010 | 1.44 (1.12, 1.85) | 0.005 | 1.40 (1.12, 1.74) | 0.003 | TBMMI, age, sex, MELD-Na, GRWR |
| Mortality, ≤1 year | 23 | 1.27 (1.01, 1.59) | 0.042 | 1.29 (1.03, 1.61) | 0.025 | 1.27 (1.06, 1.53) | 0.010 | TBMMI, age, MELD-Na |
| Mortality, ≤3 years | 44 | 1.18 (1.00, 1.40) | 0.048 | 1.19 (1.01, 1.41) | 0.043 | 1.20 (1.06, 1.35) | 0.004 | TBMMI, age |

Hazard ratios are expressed per 1 kg/m² lower TBMMI. All models are Cox proportional hazards models fitted in the whole cohort ($n$ = 802). Prespecified model: TBMMI, recipient age, recipient sex, MELD-Na, graft-to-recipient weight ratio, and massive transfusion.

Extended model: the prespecified covariates plus hypertension, diabetes mellitus, donor age, and donor sex.

Backward elimination: stepwise backward selection from the extended candidate set; the covariates retained in the final model for each outcome are listed in the last column.

Abbreviations: CI, confidence interval; GRWR, graft-to-recipient weight ratio; HR, hazard ratio; MELD-Na, Model for End-Stage Liver Disease–Sodium; TBMMI, total body muscle mass index.

**Table S7.** Association between TBMMI and graft failure according to renal function and liver disease severity.

| Subgroup | *N* | Events | Adjusted HR (95% CI)* | *p* for interaction† |
|---|---|---|---|---|
| *Renal function* | | | | |
| eGFR < 60 mL/min/1.73 m² | 46 | 3 | 1.10 (0.66–1.84) | 0.743 |
| eGFR ≥ 60 mL/min/1.73 m² | 756 | 47 | 1.20 (1.02–1.42) | |
| *Liver disease severity* | | | | |
| MELD-Na < 15 | 538 | 23 | 1.09 (0.88–1.34) | 0.265 |
| MELD-Na ≥ 15 | 264 | 27 | 1.25 (1.04–1.50) | |

Cox proportional hazards models for graft failure with follow-up administratively truncated at 3 years (802 recipients, 50 events). Estimated glomerular filtration rate was calculated with the 2021 CKD-EPI creatinine equation from the preoperative serum creatinine concentration.

*Hazard ratio per 1 kg/m² lower TBMMI, estimated within each stratum from a single model containing a multiplicative interaction term between TBMMI and the subgroup variable. Models were adjusted for recipient age, recipient sex, the MELD-Na score, the graft-to-recipient weight ratio, and massive transfusion—the same covariates as the prespecified model; in the models stratified by liver disease severity the continuous MELD-Na term was replaced by the subgroup variable.

†From a likelihood-ratio test comparing the interaction model with the corresponding model without the interaction term.

Abbreviations: CI, confidence interval; eGFR, estimated glomerular filtration rate; MELD-Na, Model for End-Stage Liver Disease–Sodium; TBMMI, total body muscle mass index.

**Table S8.** Assessment of the proportional hazards assumption and of the functional form of continuous covariates.

| | Graft failure | | All-cause mortality | |
|---|---|---|---|---|
| Model diagnostic | Statistic | *p* | Statistic | *p* |
| *(a) Proportional hazards assumption (Schoenfeld residual test)* | | | | |
| TBMMI | 6.60 | 0.010 | 4.29 | 0.038 |
| Recipient age | 0.01 | 0.942 | 1.61 | 0.204 |
| Recipient sex | 2.23 | 0.136 | 1.18 | 0.277 |
| MELD-Na | 6.65 | 0.010 | 3.59 | 0.058 |
| Graft-to-recipient weight ratio | 0.30 | 0.584 | 0.00 | 0.997 |
| Massive transfusion | 2.66 | 0.103 | 0.93 | 0.335 |
| Global test (6 degrees of freedom) | 13.52 | 0.036 | 10.30 | 0.112 |
| *(b) Functional form: linear term versus natural cubic spline** | | | | |
| TBMMI | 0.66 | 0.719 | 0.33 | 0.847 |
| Recipient age | 1.31 | 0.518 | 1.58 | 0.455 |
| MELD-Na | 10.96 | 0.004 | 9.57 | 0.008 |
| Graft-to-recipient weight ratio | 0.74 | 0.690 | 0.42 | 0.810 |
| *(c) Time-varying effect of TBMMI (interaction with log time)* | | | | |
| TBMMI × log(time), hazard ratio | 1.07 | 0.081 | 1.06 | 0.282 |
| *(d) TBMMI estimate with MELD-Na modeled as a natural cubic spline* | | | | |
| Hazard ratio per 1 kg/m² lower TBMMI (95% CI) | 1.20 (1.03–1.41) | | 1.20 (1.01–1.42) | |

All diagnostics were performed on the prespecified multivariable Cox models with follow-up administratively truncated at 3 years, containing TBMMI, recipient age, recipient sex, MELD-Na, graft-to-recipient weight ratio, and massive transfusion ($n$ = 802; 50 graft failures and 44 deaths). In panel (a) the proportional hazards assumption was assessed with the Schoenfeld residual test; each covariate is tested on 1 degree of freedom.

*In panel (b) each covariate was refitted as a natural cubic spline with 3 degrees of freedom and compared with the corresponding linear term by likelihood-ratio test (2 degrees of freedom); a significant test indicates departure from linearity.

In panel (c) the hazard ratio is the coefficient of the interaction between TBMMI and log(time), fitted as a time-transform term; a value above 1 indicates weakening of the association with increasing time since transplantation.

In panel (d) the prespecified model was refitted with MELD-Na as a natural cubic spline with 3 degrees of freedom; hazard ratios are expressed per 1 kg/m² lower TBMMI.

Abbreviations: CI, confidence interval; MELD-Na, Model for End-Stage Liver Disease–Sodium; TBMMI, total body muscle mass index.

**Table S9.** Incremental prognostic value of TBMMI and SMI for graft failure.

| Model | HR (95% CI) | LRT *p*† | *C*-index | Δ*C*-index‡ | *p*‡ |
|---|---|---|---|---|---|
| Base model | — | — | 0.657 | — | — |
| Base + SMI | 1.07 (1.02–1.12) | 0.003 | 0.677 | +0.019 | 0.423 |
| Base + TBMMI | 1.25 (1.06–1.47) | 0.009 | 0.681 | +0.024 | 0.238 |

Cox proportional hazards models for graft failure with follow-up administratively truncated at 3 years (50 events among 802 recipients). Base model: age, sex, body mass index, and MELD-Na. Hazard ratios are expressed per 1 kg/m² lower value of the index.

†Likelihood-ratio test versus the base model (1 degree of freedom).

‡Change in Harrell C-index versus the base model, with the *p* value from a test for two correlated concordance estimates. (Base + TBMMI vs. Base + SMI: ΔC-index +0.004, $p = 0.84$)

Abbreviations: CI, confidence interval; HR, hazard ratio; LRT, likelihood-ratio test; MELD-Na, Model for End-Stage Liver Disease–Sodium; SMI, skeletal muscle index; TBMMI, total body muscle mass index.
